# Central Application of Aliskiren, a Renin Inhibitor, Improves Outcome After Experimental Stroke Independent of Its Blood Pressure Lowering Effect

**DOI:** 10.3389/fneur.2019.00942

**Published:** 2019-09-04

**Authors:** Hamdollah Panahpour, Nicole A. Terpolilli, David Schaffert, Carsten Culmsee, Nikolaus Plesnila

**Affiliations:** ^1^Laboratory of Experimental Stroke Research, Institute for Stroke and Dementia Research (ISD), Munich University Hospital, Munich, Germany; ^2^Department of Physiology, Medical School, Ardabil University of Medical Sciences, Ardabil, Iran; ^3^Department of Neurosurgery, Munich University Hospital, Munich, Germany; ^4^Munich Cluster for Systems Neurology (SyNergy), Munich, Germany; ^5^Pharmaceutical Biotechnology, Department of Pharmacy, Ludwig-Maximilians University, Munich, Germany; ^6^Institute for Pharmacology and Clinical Pharmacy, University of Marburg, Marburg, Germany

**Keywords:** brain edema, focal Ischemia, neuroprotection, stroke, acute stroke, hypertension, experimental

## Abstract

Epidemiological studies suggest that pharmacological reduction of systemic hypertension lowers incidence and severity of stroke. However, whether the reduction of blood pressure *per se* or the compounds used to reduce hypertension are responsible for this effect received little attention. In the current study we therefore aimed to investigate whether Aliskiren, a renin-inhibitor used to treat arterial hypertension, may improve outcome in a mouse model of ischemic stroke when applied centrally and in a dose not affecting blood pressure. Male C57BL/6 mice received 0.6, 2.0, or 6.0 μg Aliskiren or vehicle by intracerebroventricular injection as a pre-treatment and were then subjected to 60 min of middle cerebral artery occlusion (MCAo). Infarct volume, brain edema formation, mortality, antioxidant effects, and functional outcome were assessed up to seven days after MCAo. Central administration of Aliskiren (0.6 or 2.0 μg) had no effect on systemic blood pressure but significantly reduced infarct volume and brain edema formation, blunted mortality, and improved neurological outcome up to 1 week after MCAo. Due to the central and prophylactic administration of the compound, we cannot make any conclusions about the potency of Aliskiren for acute stroke treatment, however, our study clearly demonstrates, that in addition to lowering blood pressure Aliskiren seems to have a direct neuroprotective effect. Hence, renin-inhibitors may be an effective addition to prophylactic treatment regimens in stroke patients.

## Introduction

Epidemiological studies suggest that treating arterial hypertension reduces the incidence and the severity of ischemic and hemorrhagic stroke (1, 2). This effect is attributed to the reduction of hypertension, however, an alternative explanation could be that pharmacological compounds used to reduce systemic blood pressure may in addition also have neuroprotective effects.

One pathway that has been implicated in the pathophysiology of hypertension and ischemic stroke, is the renin-angiotensin-system (RAS) (3). The RAS plays a crucial role in the maintenance of blood pressure and blood volume. Drops in blood pressure or blood volume lead to secretion of renin, a protease, which hydrolyses angiotensinogen to angiotensin I (Ang I). Ang I is converted to Angiotensin II (Ang II), a strong vasoconstrictor, by angiotensin converting enzyme (ACE). Ang II activates Angiotensin 1 receptors (AT_1_R), which induce vasoconstriction, inflammatory changes and oxidative stress, and Angiotensin 2 receptors (AT_2_R) which mediate vasodilation via Angiotensin 1-7 (Ang1-7) and MAS receptors, the so called “alternative axis” (4).

Accordingly, the RAS reduces blood pressure via inhibition of AT_1_ receptors and/or activation of AT_2_ receptors, but may well deteriorate ischemic damage thorough vasoconstriction and the other actions of AT_1_ receptors (4). In fact, inhibition of ACE or angiotensin type 1 receptors were shown to reduce arterial hypertension and to effectively prevent cerebro-vascular events (5, 6), while also being protective after cerebral ischemia. Further, activation of the AT_2_ axis inferred neuroprotection after experimental stroke (7–9).

Despite this elegant work on the downstream members of the RAS, relatively little is known about the role of renin after stroke, the first step of the RAS-cascade, which is the rate-limiting enzyme of the whole system (10–12). The activity of renin can be inhibited by the small molecule Aliskiren, a clinically frequently used compound, which was shown to effectively reduce arterial hypertension (13–16), and its sequels, that is nephropathy (17, 18) and myocardial infarction (19, 20). With regard to cerebral ischemia, however, it is not known whether Aliskiren prevents stroke only by its blood pressure lowering effect or possibly also by a direct neuroprotective effect on the brain. To investigate this hypothesis we applied not blood pressure lowering doses of Aliskiren by intracerebroventricular injection to mice, subjected them to experimental stroke, and investigated infarct volume, brain edema formation, and neurological function for up to seven days thereafter.

## Materials and Methods

All protocols used were in accordance with international guidelines, the Basel Declaration, and approved by the Government of Upper Bavaria (protocol number 55.2-1-54-2531-118-05). Results are reported according to the ARRIVE guidelines (21). Mice were randomly assigned to experimental groups by drawing lots. Surgical preparation, physiological monitoring, and data analysis were performed by an investigator (H.P.) blinded to the treatment of the animals.

## Transient Cerebral Ischemia

Cerebral ischemia was induced by middle cerebral artery occlusion (MCAo) as previously described (22, 23). Briefly, after induction of isoflurane anesthesia (induction: 30 s at 4%, continuation with 1.0–1.2% in 30% O_2_ and 70% N_2_O), a silicone covered 8-0 monofilament (Ethilon^®^, Ethicon, Germany) was advanced intravascularly via the left common carotid artery and the left internal carotid artery (ICA) until it occluded the middle cerebral artery at the MCA bifurcation. Cerebral blood flow was continuously and non-invasively measured over the left MCA territory via Laser Doppler flowmetry (LDF, Perimed, Sweden) during the procedure. MCAo was confirmed by a drop of CBF to <20% of baseline. The filament was then fixed and animals were allowed to wake up from anesthesia. Later on animals were re-anesthetized and the filament was removed 60 min after the initiation of ischemia. Animals were kept in an incubator heated to 32°C for 6 h after surgery to avoid hypothermia.

## Quantification of Infarct Volume

Twenty four hours or seven days after reperfusion mice were sacrificed in deep isoflurane anesthesia and brains were removed and frozen on powdered dry ice. Twelve sequential 10 μm coronal Nissl sections were prepared every 500 μm. Infarct volume was calculated as previously described by histomorphometry using a digital image analysis system (Olympus DP–Soft, Munich, Germany) (23–25). Infarct volumes were corrected for brain swelling and were calculated for the whole brain as well as for subcortical and cortical brain regions.

## Measurement of Brain Edema

Brain water content was assessed as previously described (26): after brain removal 24 h after reperfusion olfactory bulb, cerebellum, and medulla oblongata were dissected, cerebral hemispheres divided and weighed (wet weight, WW). After drying the tissue at 110°C for 24 h the dry weight (DW) was obtained. Brain water content was calculated using the following formula: (WW–DW)/WW × 100.

## Evaluation of Neurological Deficits

All neurological testing was performed by a researcher blinded to treatment type and group. Neurological deficits were assessed immediately before and 24 h after MCAo using a modified 6 point scale (Neurological deficit score, NDS) assessing spontaneous motor function and tail suspension; in the chronic group the testing was repeated every 24 h. Scoring was performed as described: 1 = Normal motor function (spontaneous, tail suspension), 2 = Normal spontaneous motor function, flexion of contralateral forelimb during tail suspension, 3 = Circling during tail suspension, 4 = Spontaneous circling, 5 = Leaning to contralateral side, loss of righting reflex, 6 = No spontaneous motor activity.

## Drug Administration

The specific renin inhibitor Aliskiren was extracted from a commercially available formulation (Novartis, Basel, Switzerland), purified to >99%, and administered at a concentration of 0.6, 2.0, or 6.0 μg in 2 μl saline 45 min before induction of MCAo by intracerebroventricular (i.c.v.) administration (0.9 mm left and 0.1 mm posterior to the bregma; depth: 3.1 mm). Vehicle treated animals received 2 μl of saline (vehicle).

## Assessment of Antioxidant Enzyme Activities and Lipid Peroxidation

Twenty four hours after induction of ischemia, animals were sacrificed. The ischemic hemisphere was dissected, weighed and homogenized (1:6) in phosphate buffered saline (pH 7.4) with 0.1 mM EDTA. After centrifugation at 10,000 g for 15 min at 4°C, the resulting supernatant was used for analysis. The concentration of malondialdehyde (MDA)was determined using a colorimetric assay kit (# 10009055, Cayman Chemicals, USA). The enzyme activities of superoxide dismutase (SOD, #706002), catalase (CAT, #707002), and glutathione peroxidase (GPX, #703102) in brain tissue homogenate were measured using respective assay kits (Cayman Chemicals, USA).

## Experimental Groups

**Figure 1:**
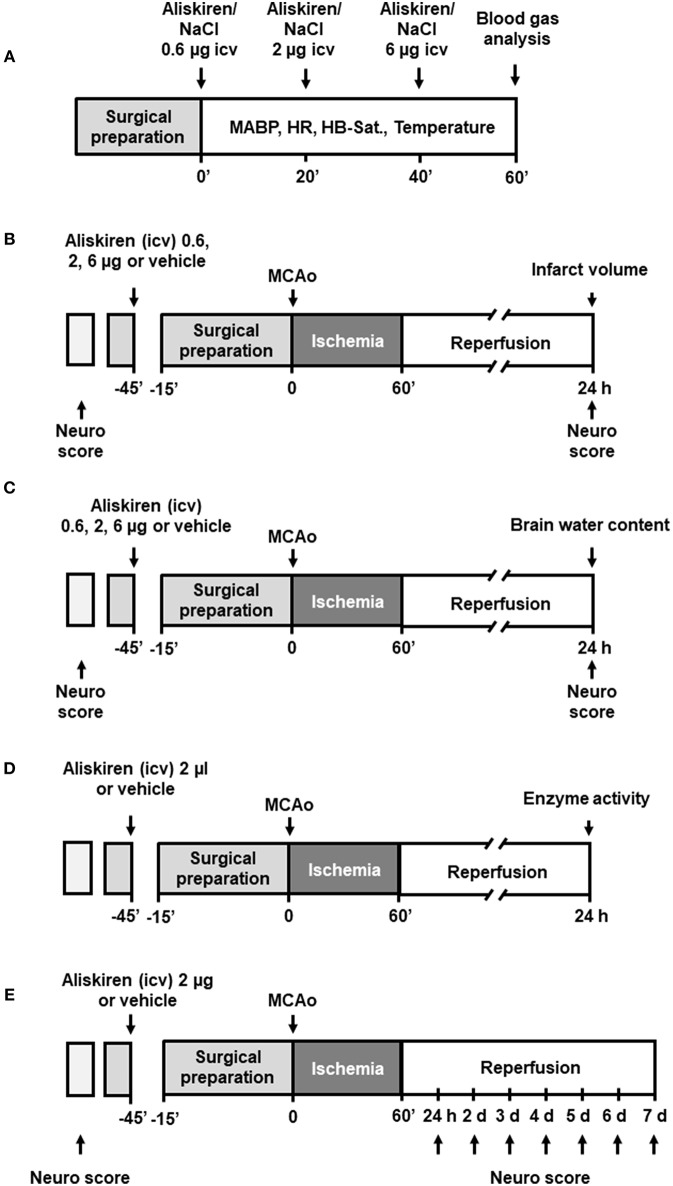
Experimental setup. **(A)** Dose finding study. **(B)** Assessment of infarct volume and **(C)** brain water content 24 h after MCAo. **(D)** Measurement of antioxidant enzyme activity 24 h after MCAo. **(E)** Evaluation of outcome as assessed by Neurological Deficit Score for 7 days after MCAo.

For dose finding (see Figure 1A for schematic drawing of the experimental setup) Aliskiren was injected i.c.v. in incrementing doses every 20 min (0.6, 1.4, and 4 μg drug in 2 μl physiological saline) to achieve the cumulative doses of 0.6, 2, and 6 μg in CSF; the control group received an equal volume of saline. Mean arterial blood pressure (MAP, measured using a femoral artery catheter), oxygen saturation, heart rate (measured by an oxygen saturation sensor at the hind paw), and body temperature (rectal temperature probe) were continuously measured. At the end of each experiment arterial blood samples were collected for blood gas analysis. Infarct volumes and neurological deficits were investigated 24 h after 60 min MCAo in four groups (*n* = 7 mice each; see Figure 1B): (1) Vehicle, (2) 0.6 μg Aliskiren, (3) 2 μg Aliskiren, (4) 6 μg Aliskiren. Treatment was administered i.c.v. 45 min before induction of MCAo. Brain edema formation and neurological outcome (Figure 1C) were assessed 24 h after 60 min MCAo in the following groups (*n* = 7 each): (1) Sham, (2) MCA occlusion only, (3) MCAo and Vehicle, MCAo and (4) 0.6 μg Aliskiren, (5) 2 μg Aliskiren, and (6) 6 μg Aliskiren; again, injections were administered 45 min before induction of MCAo. As both groups were operated under the same conditions, the results for neurological outcome are summarized (*n* = 7). Lipid peroxidation was assessed 24 h after MCAo induction in animals that received 2 μl Aliskiren or vehicle 45 min before induction of ischemia (*n* = 7 each, Figure 1D). Lastly, neurological function and bodyweight were assessed before and every 24 h after 60 min of MCA occlusion over 7 days; 45 min before induction of MCAo, Aliskiren (2 μg) in 2 μl or 2 μl saline were injected i.c.v. (*n* = 8 each group, Figure 1E).

## Statistical Analysis

Sample size calculations were performed using SigmaStat (Version 13.0, Jandel Scientific, Erkrath, Germany) with the following parameters: alpha error = 0.05, beta error = 0.2, calculated standard deviation ranged from 15 to 20% (depending on the parameter investigated), and a biologically relevant difference of 30%. After performing normality and an equal variance tests the Mann-Whitney rank sum test was used for comparisons between two groups and Analysis of Variance (ANOVA) on ranks was performed for multi group comparisons. Differences between groups were considered significant at *p* < 0.05. All data are presented as mean ± standard error of the mean (SEM), if not indicated otherwise.

## Results

### Dose Finding

**Figure 2:**
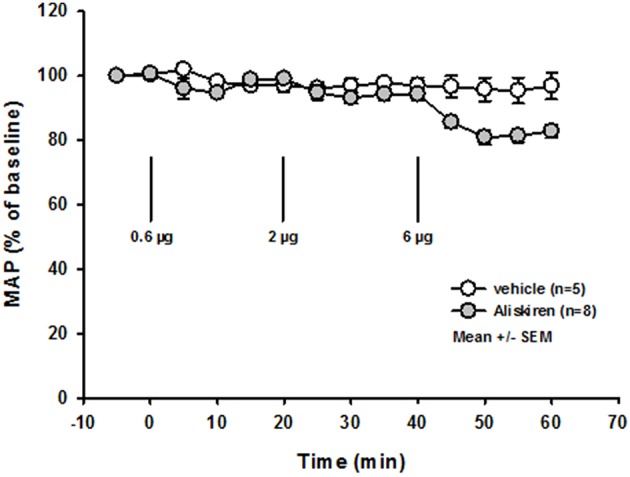
Dose finding. Mean arterial blood pressure is not affected by injection of increasing doses of Aliskiren; after a cumulative dose of 6 μg MAP tends to lower, therefore no higher concentration was used.

Repetitive injections of Aliskiren in increasing doses did not affect blood gas parameters (Table 1), heart rate, body temperature, or oxygen saturation (Table 2). Mean arterial blood pressure was slightly lowered after injection of a cumulative dose of 6 μg (Figure 2); higher doses (up to 20 μg, data not shown) induced a significant decrease of blood pressure. Therefore, the first set of experiments on neuroprotection were performed with a maximum dose of 6 μg.

**Table 1:** Blood gas analysis.

	**Vehicle group**	**Aliskiren**
**pH**	7.26 ± 0.04	7.31 ± 0.03
**pO**_**2**_	170 ± 10 mmHg	194 ± 5 mmHg
**pCO**_**2**_	42 ± 8 mmHg	39 ± 4 mmHg

**Table 2:** Physiological parameters during dose finding.

	**Injection 1 (0.6 μg)**		**Injection 2 (1.4 μg)**		**Injection 3 (4 μg)**	
	**Before**	**After**	**Before**	**After**	**Before**	**After**
**Heart rate (beats/min)**						
Vehicle group	615 ± 26	600 ± 14	596 ± 10	597 ± 8	594 ± 5	596 ± 5
Aliskiren	632 ± 7	622 ± 6	618 ± 5	613 ± 5	610 ± 6	595 ± 7
**Temperature (°C)**						
Vehicle group	37.3 ± 0.2	37.3 ± 0.2	37.3 ± 0.2	37.3 ± 0.2	37.3 ± 0.2	37.3 ± 0.2
Aliskiren	37.4 ± 0.1	37.3 ± 0.1	37.3 ± 0.2	37.2 ± 0.1	37.3 ± 0.1	37.3 ± 0.1
**Oxygen saturation (%)**						
Vehicle group	98.1 ± 0.4	98.7 ± 0.3	98.0 ± 0.8	97.8 ± 0.6	98.2 ± 0.6	98.7 ± 0.4
Aliskiren	97.6 ± 1.7	98.1 ± 1.0	97.7 ± 1.1	97.7 ± 1.3	97.5 ± 1.5	97.8 ± 1.1

### Ischemic Brain Damage 24 h After MCAo

**Figure 3:**
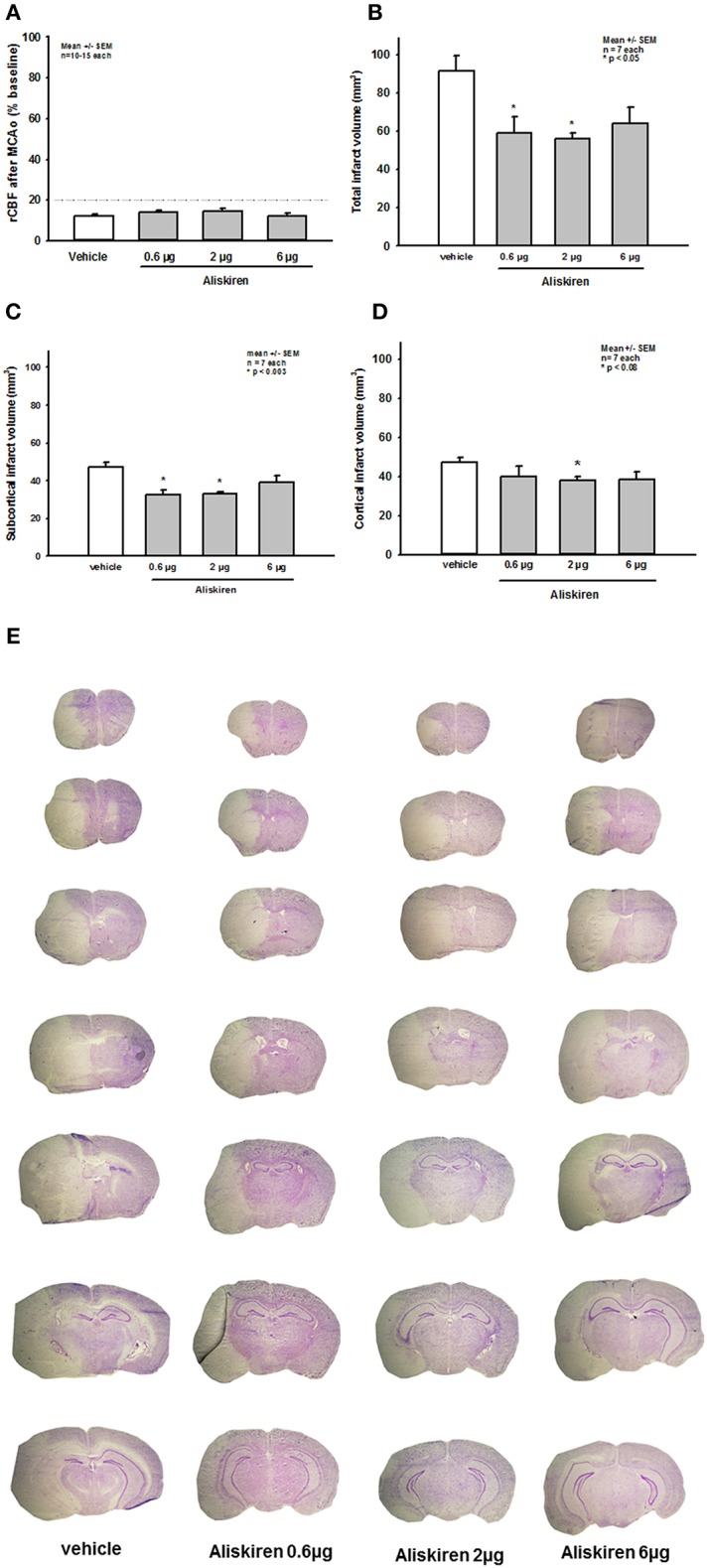
Effect of Aliskiren on post-ischemic brain damage 24 h after MCAo. **(A)** CBF after induction of MCAo. Cerebral blood flow drops to <20% of baseline immediately after filament occlusion of the MCA in all groups. **(B–D)** Infarct volumes 24 h after MCAo. **(B)** Total infarct volume is significantly reduced in the 0.6 and 2 μg Aliskiren treatment groups. **(C)** Subcortical infarct volume was also reduced in these groups while **(D)** cortical infarct volumes were only significantly smaller in the 2 μg group compared to vehicle treated animals. **(E)** Exemplary coronal Nissl sections 24 h after MCAo.

Immediately after induction of filament occlusion of the left MCA, cerebral blood flow over the left MCA territory dropped below 20% of baseline cerebral perfusion in all groups (Figure 3A). Twenty four hours after 60 min of transient MCA ischemia, total infarct volume was significantly lower in the 0.6 and 2.0 μg Aliskiren groups as compared to vehicle treated controls (Figure 3B; vehicle: 91.7 ± 7.7 mm^3^; 0.6 μg Aliskiren: 58.9 ± 8.7 mm^3^, *p* < 0.003; 2 μg Aliskiren: 56.0 ± 7.1 mm^3^, *p* < 0.003; 6 μg Aliskiren: 63.9 ± 8.5 mm^3^, *p* = 0.165). Subcortical infarct volume (Figure 3C) was significantly decreased in both the 0.6 μg as well as the 2 μg Aliskiren group (*p* < 0.003 vs. vehicle control); cortical infarct volume was only significantly lower in the 2 μg Aliskiren group (Figure 3D, *p* < 0.008). Figure 3E shows exemplary coronal brain sections for all groups.

**Figure 4:**
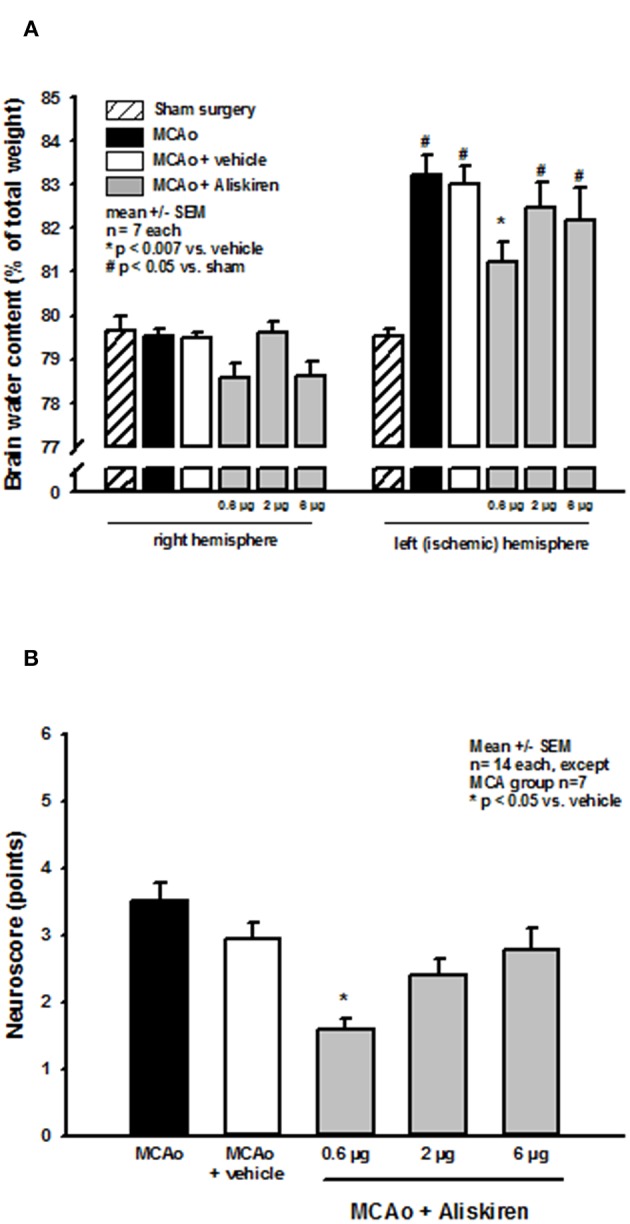
Brain edema formation and neurological outcome after renin inhibition. **(A)** Brain water content 24 h after MCAo. Brain water content in the non-ischemic right hemisphere of all MCAo groups was not altered compared to sham operated animals; all animals that underwent ischemia showed significant brain edema formation in the left, i.e., ischemic hemisphere. Brain water content was significantly reduced by application of 0.6 μg Aliskiren. **(B)** Neurological deficit score after renin inhibition. Twenty four hours after MCAo all animals had significant motor deficits; Aliskiren 0.6 μg significantly improved the neurological scores; higher doses tended to reduce the score but did not have a significant effect on the animals performance.

Brain water content in the right (non-ischemic) hemisphere (Figure 4A, left side) was not altered in the treatment or vehicle groups as compared to sham operated animals (sham) or animals that underwent MCA occlusion without i.c.v. injection (MCA only). MCA occlusion led to a significant increase of brain water content in the left hemisphere in all MCAo groups compared to sham operated animals indicating the formation of brain edema under these conditions (Figure 4A right side, *p* < 0.05 vs. sham). Brain edema formation was significantly reduced after 0.6 μg Aliskiren (*p* < 0.007 vs. vehicle); higher doses had no significant effect.

### Effect of Renin Inhibition on Post-Ischemic Neurologic Deficits

MCAo lead to significant deterioration of motor function as assessed by the neurological deficit score; mice in the 0.6 μg Aliskiren group achieved significantly better scores than vehicle treated animals (Figure 4B). Higher doses did not improve neurological outcome.

### Assessment of Lipid Peroxidation Enzyme Activity

**Figure 5:**
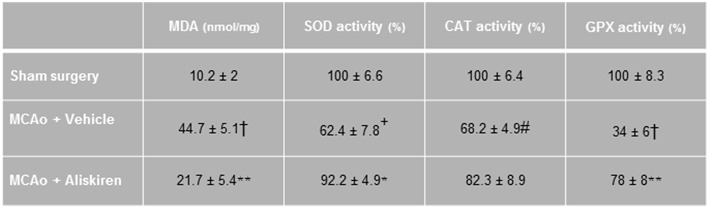
Effects of Aliskiren (2 μg) on lipid peroxidation and brain tissue antioxidant enzyme activities in brain tissue homogenate of studied groups 24 h after MCAO. MDA, malondialdehyde; SOD, superoxide dismutase; CAT, catalase; GPX, glutathione peroxidase (*n* = 7). Values are mean ± SEM. *^†^p* < 0.001, ^+^*p* < 0.01, and ^#^*p* < 0.05 compared with sham group; **p < 0.01 and *p < 0.05 compared with vehicle group.

Ischemia and reperfusion resulted in a significantly increased MDA concentration and reduced antioxidant enzyme activities in the ischemic hemispheres as compared to the sham group, consistent with enhanced lipid peroxidation after ischemic stroke. Treatment with Aliskiren significantly reduced the MDA concentration (*p* < 0.01) and elevated superoxide dismutase and glutathione peroxidase activities (*p* < 0.05 and *p* < 0.01 vs. vehicle group, respectively, Figure 5).

### Effects of Renin Inhibition on Outcome Parameters Over 7 Days After MCAo

Based on the observation that Aliskiren improved histopathological and functional outcome 24 h after experimental stroke, we wanted to evaluate if this effect lasted long-term, i.e., for at least 1 week. Since only 2 μg of Aliskiren reduced subcortical and cortical infarct volumes we decided to use this doe for these experiments.

**Figure 6:**
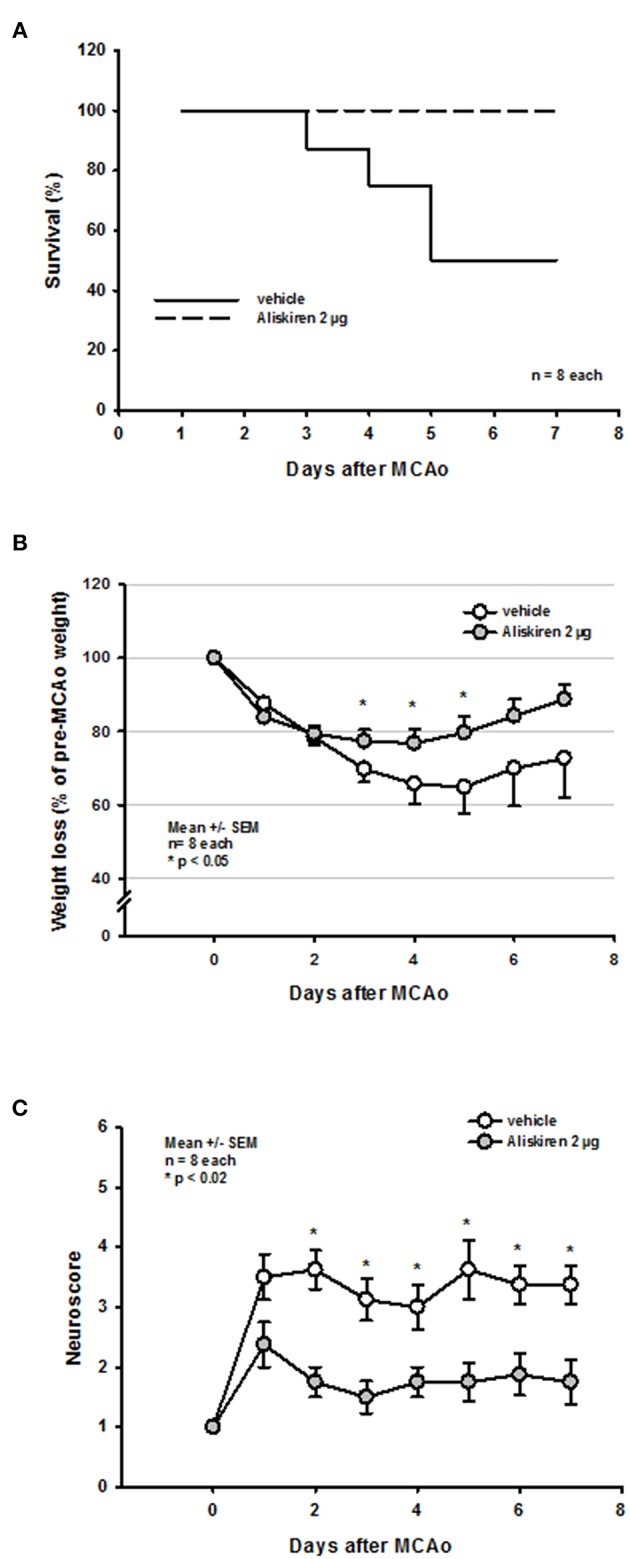
Functional outcome up to 7 days after MCAo. **(A)** While mortality in the vehicle group was 50% over time all Aliskiren animals survived. **(B)** Treatment animals recovered significantly quicker from post-ischemic weight loss. **(C)** Starting 2 days after MCAo, Aliskiren treated mice achieved significantly better scores for motor function.

Seven days after 60 min MCAo vehicle treated mice showed a mortality of 50% (Figure 6A). Interestingly, all mice receiving Aliskiren survived. The long-term protective effect of Aliskiren is also impressively exemplified by a less pronounced weight loss in the treatment group 3, 4, and 5 days after MCAo (Figure 6B) and by a significantly better functional outcome staring from day 2 after MCAo until the end of the observation period (Figure 6C).

## Discussion

Here, we report that central application of the renin antagonist Aliskiren, a clinically used antihypertensive drug, protects the brain after experimental ischemic stroke independent of its blood pressure lowering activity. Aliskiren significantly attenuated brain edema formation and stroke volume, blunted mortality and improved neurological outcome in a murine stroke model. Hence, the clinically observed effect of Aliskiren on stroke outcome may not only be related to its effect on blood pressure, but also to a direct effect of renin on the pathophysiology of ischemia-induced brain damage.

In order to test the hypothesis of the current study, namely whether the antihypertensive drug Aliskiren may have effects on the ischemic brain beyond its actions on systemic blood pressure, we used the format of a pharmacological study. As a prerequisite for such a study type we tested multiple dosages, took care that the investigated compound reached the target organ (by applying Aliskiren by intracerebroventricular injection), carefully and extensively monitored animal physiology, and performed and analyzed all experiments in a strictly randomized and blinded fashion in order to avoid any investigator-induced bias. To test the short- and long-term effect of Aliskiren in an animal model of stroke we occluded the middle cerebral artery for 60 min using an intraluminal filament, a very well-established and standardized model in our laboratory (24, 26, 27). This was further proven by the fact that in the current study blood flow was reduced to ischemic levels (<20% of baseline) in all investigated animals. The rationale of using a transient ischemia model was that we wanted to be able to investigate the effect of Aliskiren on neurological function over 1 week. In order to induce neurological deficits, a model inducing large infarcts is necessary, which, however, is only survived by mice when the occlusion is transient. Therefore, we could not use a permanent ischemia model, which may have an improved translational profile, for the current study.

Aliskiren has been used in models of cerebral ischemia before, however, it has only been applied systemically (28, 29) and/ or in animals with arterial hypertension (29, 30). Intravenous application of Aliskiren for 5 days before MCA occlusion resulted in improved outcome and decreased mortality in severely hypertensive rats (29) and reduced infarct volume in mice (28). These experiments, however, did not clarify whether the observed neuroprotection was due to a peripheral, i.e., blood pressure lowering, or central effects of Aliskiren. This is particularly true since it is not clear whether Aliskiren crosses the blood-brain barrier and it is well-known that it has a low resorption rate (5%) (31), low bioavailablity (32) and high plasma protein binding (32) after systemic application. Therefore, little is known about the specific central effect of Aliskiren for the development of ischemic brain damage and the current study is the first one to address this issue.

Using the current approach we have proven that centrally applied Aliskiren is neuroprotective after cerebral ischemia independent of its blood pressure lowering effect. Although we did not measure cerebral renin activity, the specificity and the well-known and proven action of Aliskiren as a renin inhibitor (33) allows us to assume with a high level of confidence that the observed neuroprotective effects of Aliskiren were indeed caused by the inhibition of renin. Taking this as given, the next important question is directed toward the mechanisms undelaying the observed functional and histopathological protection of Aliskiren after cerebral ischemia.

Renin is the first and rate limiting component of the RAS. The classical function of the RAS is the regulation of blood pressure and salt/water homeostasis, however, it has also been implicated in pathogenesis and outcome of ischemic injury in various organs such as heart (34, 35) and kidney (36, 37). All components of the RAS are present in the brain and the RAS has also been closely studied in the context of cerebrovascular disease (38) and stroke (4, 39, 40). Specifically, blocking angiotensin-converting enzyme (ACE) (39–43) or AT-1 receptors (41) has been demonstrated to be neuroprotective following ischemic stroke in experimental animals and humans. In recent years, AT-2 (8, 44, 45) or MAS (7) receptor activation have been proposed as another RAS-based therapeutic approach for reducing post-ischemic brain damage after stroke. Therefore, the most straightforward mechanistic explanation of the current results is that the inhibition of renin inhibits the more downstream members of the RAS thereby replicating their well-known neuroprotective effects. This may be a valid, but may not fully explain the quite pronounced neuroprotective effects of Aliskiren. The reason is that inhibition of renin may indeed inhibit the formation of Angiotensin-1 (Ang-1), the subsequent production of Ang-2 by ACE, and activation of damaging AT-1 signaling, however, it would also inhibit neuroprotective AT-2 and MAS signaling. Therefore, reduction of Ang-1 production may not fully explain the neuroprotection currently observed with Aliskiren and suggests that renin may have additional functions. In previous studies using Aliskiren, the protective effects of renin inhibition were hypothesized to be caused by a reduction of apoptotic mechanisms as well as anti-inflammatory effects, however, the link between renin activity and apoptotic signaling remains quite speculative (28, 29). In the present study, we found evidence for a reduction of ischemia-induced lipid peroxidation and less activity impairment of antioxidant enzymes superoxide dismutase and glutathione peroxidase. This is in keeping with previous data obtained by Aliskiren in other organs and models (46–48). As oxidative damage and lipid peroxidation play an important role in the development of postischemic damage (49–51) this finding may partially explain the neuroprotective effect observed. Another mechanism which may have a direct connection to the pathophysiology of cerebral ischemia is the link between renin and the activity of endothelial nitric oxide synthase (eNOS). This link was established since Aliskiren has been described to increase eNOS activity (52) and phosphorylation (53) and its protective effects were eNOS dependent (19) after myocardial ischemia. It is therefore quite possible that the protection observed in the current study is, at least partially, due to restoration of eNOS activity, specifically since the activity of eNOS is significantly reduced after ischemic stroke (54, 55) and increasing the formation of NO has been shown to be neuroprotective after ischemic stroke (26, 54, 56–58). These actions of renin after stroke are quite speculative and will therefore need further experimental validation. Another point that has to be elucidated in further studies is whether the Aliskiren effect observed is different at different doses.

In summary, central application of Aliskiren, a clinically approved renin inhibitor, is neuroprotective and improves functional outcome in a model of ischemic stroke independent of its blood pressure lowering activity. The experimental approach used in the current study (pre-treatment, intracerebroventricular injection) was chosen in order to supply a proof of principle with as little confounding factors as possible and does therefore not allow any conclusion about the effect of Aliskiren for acute stroke treatment. In order to further explore the therapeutic potential of the proposed neuroprotective mechanism of central renin inhibition after ischemic stroke more studies are needed in order to explore a possible therapeutic window; also, pharmacological modification of the drug which may allow for a different route of administration are in planning. Nevertheless, the observed neuroprotection was relevant and reproducible. Therefore, central inhibition of renin may represent a promising approach to protect the brain from ischemic injury when used prophylactically. Hence, developing centrally active renin inhibitors may provide a novel approach toward the prophylactic treatment of ischemic brain injury.

## Data Availability

The raw data supporting the conclusions of this manuscript will be made available by the authors, without undue reservation, to any qualified researcher.

## Ethics Statement

All protocols used were in accordance with international guidelines, the Basel Declaration, and approved by the Government of Upper Bavaria (protocol number 55.2-1-54-2531-118-05).

## Author Contributions

HP performed all the experiments. HP, NT, and NP analyzed and interpreted the data. DS and CC extracted and purified the drug. HP, NT, and NP wrote the manuscript.

### Conflict of Interest Statement

The authors declare that the research was conducted in the absence of any commercial or financial relationships that could be construed as a potential conflict of interest.
